# Interstitial Fluid Colloid Osmotic Pressure in Healthy Children

**DOI:** 10.1371/journal.pone.0122779

**Published:** 2015-04-08

**Authors:** Hans Jørgen Timm Guthe, Marianne Indrebø, Torbjørn Nedrebø, Gunnar Norgård, Helge Wiig, Ansgar Berg

**Affiliations:** 1 Department of Pediatrics, Haukeland University Hospital, Bergen, Norway; 2 Department of Clinical Medicine, University of Bergen, Bergen, Norway; 3 Department of Clinical Medicine, Faculty of Medicine, University of Oslo, Oslo, Norway; 4 Department of Biomedicine, University of Bergen, Bergen, Norway; 5 Department of Occupational Medicine, Hyperbaric Medical Unit, Haukeland University Hospital, Bergen, Norway; 6 Department of Clinical Medicine, Faculty of Medicine, Section for Pediatric heart-, lung- and allergic diseases, University of Oslo, Oslo, Norway; 7 Department of Clinical Science, University of Bergen, Bergen, Norway; Kurume University School of Medicine, JAPAN

## Abstract

**Objective:**

The colloid osmotic pressure (COP) of plasma and interstitial fluid play important roles in transvascular fluid exchange. COP values for monitoring fluid balance in healthy and sick children have not been established. This study set out to determine reference values of COP in healthy children.

**Materials and Methods:**

COP in plasma and interstitial fluid harvested from nylon wicks was measured in 99 healthy children from 2 to 10 years of age. Nylon wicks were implanted subcutaneously in arm and leg while patients were sedated and intubated during a minor surgical procedure. COP was analyzed in a colloid osmometer designed for small fluid samples.

**Results:**

The mean plasma COP in all children was 25.6 ± 3.3 mmHg. Arbitrary division of children in four different age groups, showed no significant difference in plasma or interstitial fluid COP values for patients less than 8 years, whereas patients of 8-10 years had significant higher COP both in plasma and interstitial fluid. There were no gender difference or correlation between COP in interstitial fluid sampled from arm and leg and no significant effect on interstitial COP of gravity. Prolonged implantation time did not affect interstitial COP.

**Conclusion:**

Plasma and interstitial COP in healthy children are comparable to adults and COP seems to increase with age in children. Knowledge of the interaction between colloid osmotic forces can be helpful in diseases associated with fluid imbalance and may be crucial in deciding different fluid treatment options.

**Trial Registration:**

ClinicalTrials.gov NCT01044641

## Introduction

Maintenance of body fluid homeostasis requires a delicate balance between the hydrostatic and colloid osmotic pressure (COP) acting across the intravascular and interstitial compartments. According to the classical Starling hypothesis, the net fluid shift across the capillary membrane is based on the interaction between two opposing forces: the difference in hydrostatic pressures and COP on either side of the membrane separating the capillary and the interstitial fluid (IF) spaces [1]. Studies of these parameters under various conditions with fluid retention (e.g. nephrotic syndrome [2], premenstrual syndrome [3], normal pregnancy [4] and cardiopulmonary disease [5]) in humans have had great significance for understanding the pathophysiology of these disorders, and have in some cases been important in the choice of fluid treatment [5]. The normal values for colloid osmotic and hydrostatic pressures in the resting state of healthy children have not been established, largely because of methodological difficulties in measurements of each of the parameters. In the case of interstitial colloid osmotic pressure (COP_i_), this is likely due to lack of generally accepted methods for isolating IF. What is clear, however, is that plasma colloid osmotic pressure (COP_p_) increases during the first months after birth, reaching at one year of age, values comparable to those reported in adult subjects [6]. Furthermore, decreased COP_p_ in disease states, like congenital analbuminemia and during surgery for congenital cardiac malformations, is associated with compromised pulmonary function and tissue edema [7, 8]. A commonly accepted method for IF sampling and thus COP_i_ determination is implantation of wicks [9, 10] within the proximity of the heart assuming that this position represents the average capillary pressure [11]. Gravity is thought to influence the transcapillary pressures measured below the heart level with decreased interstitial COP from an increased hydrostatic pressure gradient [11]. Harvesting IF has traditionally been managed by extraction of fluid from subcutaneously implanted nylon wicks [9]. Although evaluation studies have shown that an implantation time of 60 minutes is appropriate in adults, this supposition is not necessarily true for the pediatric population. Establishing normal values of COP_i_ in healthy children can be vital for proper fluid therapy in critically ill children. The aim of this study was primarily to evaluate the relationship between COP_p_ and COP_i_ in healthy children of different age. Secondly, we wanted to test if gravity would induce a difference in COP_i_ obtained from arm and leg and thirdly to evaluate implantation times of wicks of 60 and 90 minutes.

## Materials and Methods

The study was designed as a non-blinded, sequential, descriptive study, taking place between 05. January 2007 and 22. January 2012. Patient enrolment began before study trial was registered (ClinicalTrials.gov Identifier: NCT01044641, https://clinicaltrials.gov/ct2/show/NCT01044641?term=guthe&rank=2) due to the authors not being aware of the need for registration when the study was initiated. The authors confirm that all ongoing and related trials for this intervention are registered. The protocol was approved 29. September 2006 by the local ethics committee (Regional Committee for Medical and Health Research Ethics, Western Norway) and conducted at the outpatient clinic, Department of Ear-Nose-Throat, Haukeland University Hospital. Patients were also recruited from the Department of Ear-Nose-Throat, Akershus University Hospital from May 2010 to June 2010 to increase number of participants. Ethics approval was not necessary from Akershus University Hospital because of the existing approval from the Regional Committee for Medical and Health Research Ethics, Western Norway. For CONSORT checklist, Ethical Confirmation and Trial Protocol in Norwegian and English; see S1 CONSORT Checklist, S1 Ethics approval, S1 Protocol and S2 Protocol. The time period for patient recruitment was prolonged after approval from the local ethics committee, due to delayed enrolment of patients.

Patients were included in the study, after written informed consent was obtained from the parents or guardian. All subjects were recruited from otherwise healthy patients, 99 children (aged between 2 and 10 years), who were scheduled either for tonsillectomy and/or adenotomy and/or tympanic paracentesis. Enrolled subjects were excluded if they had any sign or significant medical history of acute febrile illness, underlying chronic diseases like cardiac anomalies, liver disease, nephropathy or any disease states and present medication that could interfere with protein metabolism. Age, gender and weight were plotted in pediatric national growth charts according to Juliusson [12].

Experiments were performed after a fasting period of at least 8 hours (no food, or chewing gum allowed after midnight the day on admission, only small amounts of water were allowed 2 hours before operation). Induction and maintenance of anesthesia consisted of weight related doses of sodium thiopental/propofol, fentanyl/remifentanil, morphine, atropine and mivacurium chloride (sevoflurane gas induction was used initially if intravenously cannulation failed). Rectal administration of paracetamol/paracetamol-kodein and non-steroidal anti-inflammatory drugs (NSAIDs) was performed after intubation, and controlled mechanical ventilation was adjusted according to end-expiratory carbon dioxide concentration and pulse oximetry. Maintenance fluid of Ringer`s solution was administered according to local guidelines during the procedure and all medication were requested and handled by anesthetists who were not involved in the study.

Procedures: Included patients were divided into age specific subgroups (2–3, 4–5, 6–7, 8–10 years) according to National Institute of Child Health and Human Development (NICHD) pediatric terminology where early childhood is defined from 2 to 5 years and middle childhood from 6 to 11 year [13]. After induction of anesthesia, sterilized multi-filamentous nylon wicks of approximately 5 cm were introduced in subcutis after skin disinfection and covered with adhesive plastic film in accordance with earlier wick studies [14]. Each patient had one wick implanted subcutaneously in one arm (lateral upper arm) at the level of the heart. This site was chosen since it was technically preferable to the thorax used in the original publication by Noddeland [11] and due to less and difficult accessible subcutaneous tissue of a small thorax. A second wick was placed in the medial part of one leg. All wicks were removed after 60 minutes according to optimal implantation/equilibration time in adults [15]. In an additional group, irrespective of age, where general anesthesia was expected to last for over 90 minutes, one wick was implanted in each medial part of the leg. One wick was retracted after 60 minutes and one wick after 90 minutes.

Plasma colloid osmotic pressure: Before surgery, in connection with peripheral intravenously cannula insertion, 0.5 ml venous blood was collected after light hemostasis. Since coagulating factors are known to have sparse effect on COP [16], plasma were allowed to coagulate in unheparinized tubes and serum was separated from the sample by centrifugation, 3000 rpm for 10 minutes, and immediately frozen in plastic tubes (Sarstedt, Reagiergefaße, micro tubes, 1,5ml) at -20^°^C. COP_p_ was measured directly with a colloid osmometer designed for small fluid samples [15, 17, 18] using a membrane impermeable for molecules > 30 kDa (PM-30 Amicron, Lexington, Ma, USA). Signals were amplified and recorded (Easy Graph P930, Gould Inc., USA).

Interstitial colloid osmotic pressure: After induction of anesthesia, two double threaded multi-filamentous nylon wicks were sewn into subcutaneous tissue. Methods and procedures were performed under sterile conditions and according to previous studies [15]. Blood stained wicks were discarded, and only clear and pink wicks with presumably none or low hemoglobin contamination were accepted for further evaluation [19]. COP_i_ was measured directly by a colloid osmometer as described above.

Circulating blood volume (CBV) for estimation of blood loss during surgery was calculated by the following formula estimated from a recent meta-analysis by Riley et al [20].

CBF = 75ml/kg bodyweight × kg bodyweight (1)

Serum albumin and hemoglobin concentration were analyzed in an automatic analyzer (Cobas 8000 c702, Roche Diagnostics, USA and CELL-DYN Sapphire, Abbott Diagnostics, USA) respectively, both by colorimetry.

Bedside hemodynamic monitoring (blood pressure, heart rate and SpO_2_) was performed immediately before, under and after the procedure according to local protocol.

Statistical Analysis: Data were evaluated using SigmaStat 11 (Sy Stat Software/Inc.; Germany). Results are presented as numbers with proportions (%) and means with standard deviation (SD). Statistically significance was defined as a P value < 0.05. One-way ANOVA was used for evaluating the COP_p_ and COP_i_ between the different age groups, followed by an all-pairwise Holm-Sidak multiple comparison procedure if there was found a statistical significance with the One-way ANOVA. When comparing smaller groups, not normally distributed, we used a non-parametric test (Mann-Whitney).

## Results

**Fig 1:**
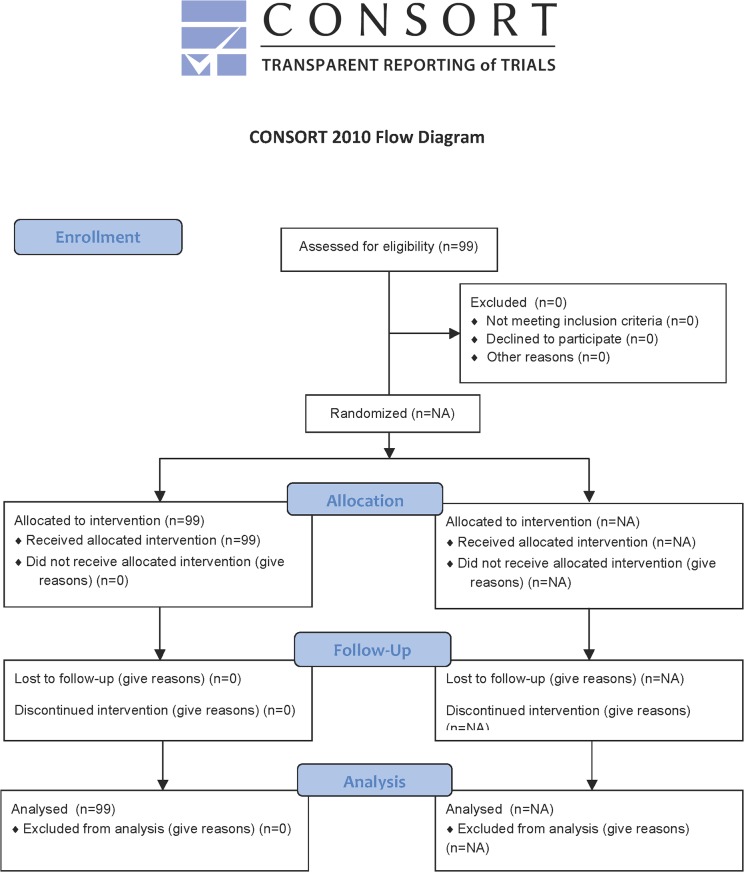
CONSORT flow diagram.

**Fig 2:**
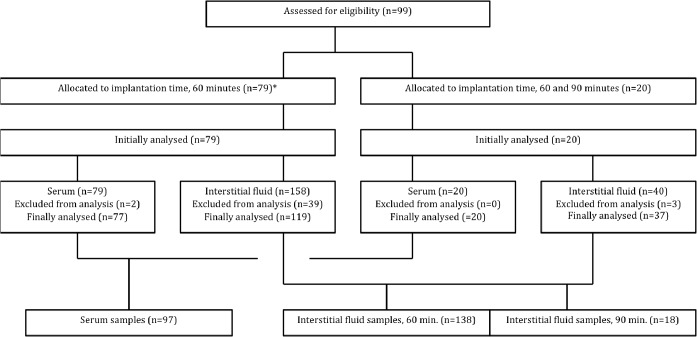
Recruitment of patients and distribution of samples. *Blood loss greater than 10% of estimated CBV (n = 11).

Patients and outcome: A total of 99 children (45 girls and 54 boys), were included in the study (Fig 1), and 92 (93%) had weights within ± 2 SD according to Norwegian growth charts (S1 Fig). Eleven participants (mean age 4 years 8 months) had blood loss greater than 10% of estimated CBV (Fig 2). Nineteen percent of wicks were discarded due too blood staining of wick or insufficient sample size (Fig 2). In average, 5 l of interstitial fluid was harvested from each wick. Preoperative hemoglobin (Hb) measured in 83 patients (84%) less than eight days before surgery averaged 12.5 g/dl (range 10.5–15.0). Serum Albumin was measured before surgery in 32 patients (32%) with mean albumin of 44 g/l (range 38–52) and no statistical difference was found between the different age groups. There were no immediate (before discharge) or long time (phone interview 7 days after the procedure) complications due to wick implantation or blood sampling.

**Fig 3:**
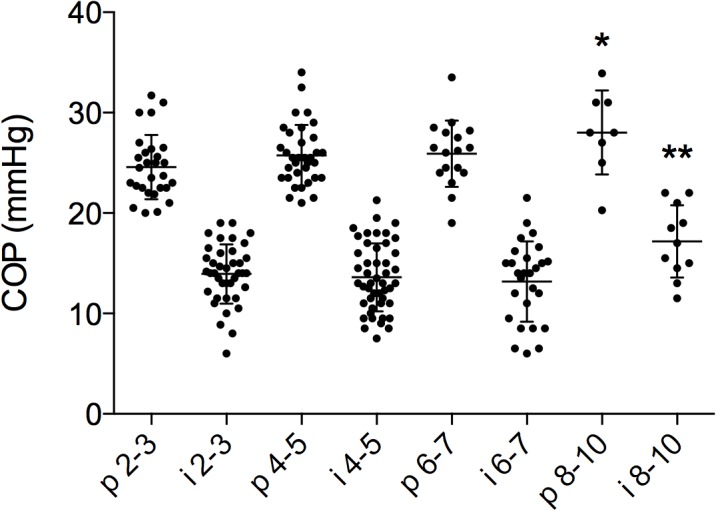
Colloid osmotic pressure in plasma and interstitium. Colloid osmotic pressure in plasma (p) and interstitium (i) (arm and leg merged) related to age. There was significant difference in pressures between 2–3 years and 8–10 years for plasma (P < 0.05, *) and between first three age groups and 8–10 years (P < 0.01, **) in interstitial fluid.

Plasma COP: Mean COP_p_ for all age groups was 25.6 ± 3.3 mmHg, and there was a significant rise from 24.6 ± 3.2 mmHg at 2–3 years to 28 ± 4.2 mmHg at 8–10 years of age, P = 0.02 (Fig 3).

**Fig 4:**
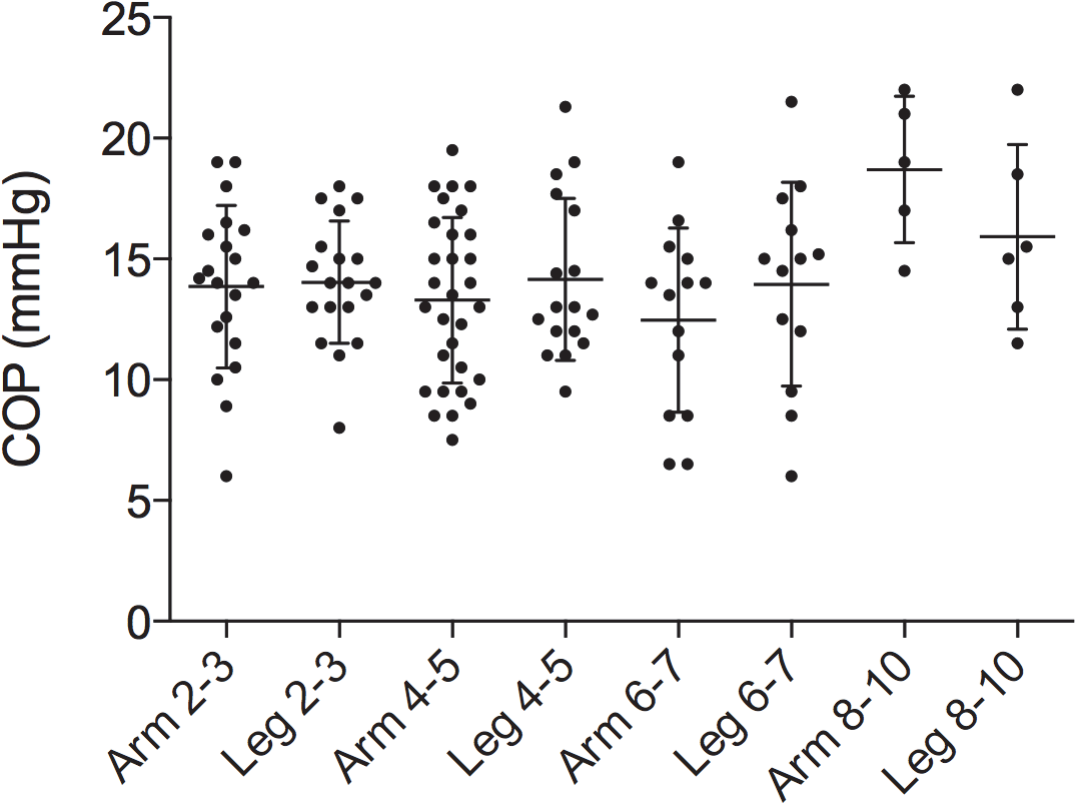
Colloid osmotic pressure in arm and leg. Colloid osmotic pressure from wicks after implantation in arm vs. leg related to age. There was no significant difference in the pressures obtained in arm and leg.

Interstitial COP: Mean COP_i_ (arm and leg together) for all children was 13.9 ± 3.5 mmHg. There were no significant differences between the 2–3, 4–5 and 6–7 year age groups, (14.2 ± 3.2 mmHg, 13.6 ± 3.4 mmHg and 13.2 ± 4.1 mmHg respectively), but the 8–10 age group had a higher value (17.2 ± 3.2 mmHg) than the younger children, P < 0.05 (Fig 3). There were no significant differences between arm and leg COP_i_ within any of the groups or between groups (Fig 4). In contrast to the other groups, the COP_i_ tended to be lower in the leg than in the arm in the 8–10 year old group, but observations were few. The mean COP_i_ (arm and leg altogether) from patients with blood loss over 10% of CBV was significantly higher than the COP_i_ obtained from the other patients (16.0 ± 4.0 mmHg vs.14.0 ± 3.6 mmHg, P = 0.048).

Twenty-two patients who received NSAIDs in combination with paracetamol had almost identical COP_i_ to those who did not receive NSAIDs (14.1 ± 3.9 mmHg vs. 14.0 ± 3.5 mmHg P = 0.9).

Eighteen patients had wicks implanted in the leg for 60 and 90 minutes. There was no significant difference in mean COP_i_ between these endpoints (11.3 ± 2.8 mmHg vs.12.9 ± 3.1 mmHg, P = 0.11).

**Fig 5:**
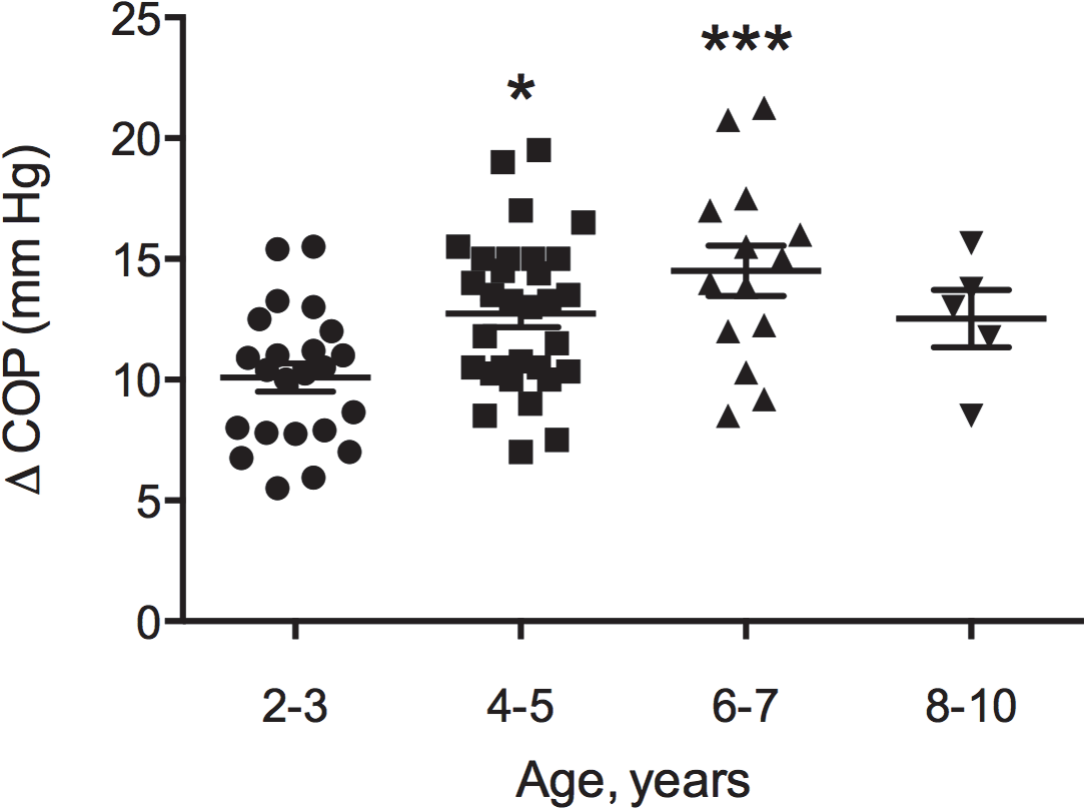
Transcapillary gradient in COP. (ΔCOP = COP_p_—COP_i_ (arm and leg merged)) ΔCOP related to age, with significant difference between 4–5 years and 2–3 years (P = 0.017 *) and between 6–7 years and 2–3 years (P < 0.001 ***).

There was a significantly increasing colloid osmotic pressure gradient between plasma and interstitium (ΔCOP) from 10.1 ± 2.8 mmHg at 2–3 years to 14.5 ± 3.9 mmHg at 6–7 years (Fig 5). The difference tended to be smaller for the 8–10 year old group (12.5 ± 2.6 mmHg), but there were only five observations.

## Discussion

In this study, COP_p_ and COP_i_ in healthy children were close to what has been reported for healthy adults [21], but both COP_p_ and COP_i_ were significantly higher at 8–10 years than in younger children. Whether COP_i_ was obtained at or below heart level, or after 60 or 90 minutes, did not influence the results. There were no complications to using nylon wicks, suggesting that this method is safe for harvesting IF in anesthetized children. Furthermore, the method also gave sufficient volumes of IF to allow COP measurements. In rats, the optimal time needed for fluid and protein transport into the wick with a minimum of inflammation is between 30 and 120 minutes [19]. As suggested for healthy adults [14, 15], we found that 60 minutes was sufficient implantation time for collection of IF.

Earlier studies by Noddeland showed a significant higher COP_i_ in the thorax wall than in the calf close to the ankle in adults who were examined both in the upright and horizontal position, and that the duration of horizontal positioning did not change COP_i_ significantly up to 40 hours [11]. We did not observe such difference in our population although the children had been freely ambulatory until shortly before the relatively minor surgical procedures. The duration of horizontal position was not recorded, but lasted at least 1 hour before sampling. Our finding of no significant difference between arm and leg COP_i_ suggests that children do not experience the same orthostatic effects on COP_i_ as adults. This conclusion is underscored by the observation that the COP_i_ in the leg was lower than in the arm for the age group closest to adulthood, but not in the younger children. The difference between adults and children may have several explanations, e.g. a larger orthostatic effect due to a greater height and poorer venous drainage as a consequence of the characteristics of the veins or less physical activity.

In accordance with earlier data of COP_i_ in ankle [11], this finding indicates that duration of implantation within these limits did not cause sufficient trauma and subsequent inflammation to produce changes in interstitial protein distribution, which may have been expected to increase with increased implantation time [22].

COP_p_ in healthy full-term (19.4 ± 2.2 mmHg) [23] and pre-term (15.4 ± 1.3 mmHg) [24] babies have been reported to be significantly lower than in healthy infants from 1 to 9 months of age [6], who may have values almost identical to that of adults (25 mmHg) [21]. Due to lack of data on changes in COP in infants up to 2 post-natal months, we anticipate a sharp increase in COP to occur within the first months of life. Our findings of COP_p_ and COP_i_ similar to what has been reported for patients from two years of age are in line with studies beyond the neonatal period, suggesting that the major change occurs at around 1–2 months of age, although our study documents that a small, but significant increase also occurs during early childhood. This rise of COP_p_ is probably due to increasing serum concentration of proteins other than albumin, which is known to occur with increasing age [25] since we experienced a concomitant elevation in both COP_p_ and COP_i_ and no age dependent change in preoperative serum albumin.

COP_p_ in healthy subjects is predominantly dependent on plasma albumin and to a lesser extent on the total plasma protein concentration [26]. Albumin concentrations in the premature infant are lower than in term newborns [27], increase gradually up to 1 years of age and then undergo a modest rise towards adulthood [25]. The apparent parallel increase in COP_p_ and albumin concentration, therefore, suggests that albumin is part of the cause of the age dependent COP_p_.

The use of hyperoncotic albumin in diseased states for fluid replacement and maintenance of COP_p_ is controversial and has not proven superior to saline with respect to mortality for adults admitted to the intensive care unit needing fluid resuscitation e.g. [28, 29]. In critically ill patients with increased vascular permeability, administration of albumin may actually worsen edema due to leakage of albumin into the interstitium with concomitant elevation of COP_i_ and intensified accumulation of interstitial fluid. There is a correlation between low COP_p_ at birth and severity of respiratory distress syndrome [30] and detection of early hypoproteinemia in sick preterm babies is associated with unfavorable outcome [31].

Blunt et al found a reduced contribution of albumin from 80% to 17% in relation to COP_p_ in sick patients [32] with no proven effect of albumin administration on mortality. Although there is a reasonable link between COP_p_ and albumin these findings may advocate reduced serum albumin simply as a marker of serious disease rather than an indicator of decreased COP_p_. There are few studies addressing albumin for fluid resuscitation in the pediatric population, and albumin is now less used as standard plasma expander in infants although potentially beneficial for children undergoing cardiopulmonary bypass [8].

Accurate measurement of CBV in children is difficult due to lack of a “gold” standard method. However, a recent meta-analysis of values from many small studies showed that a CBV of 75 ml/kg appears to be normal for children from 2 years of age [20]. A blood loss between 10% to 15% of estimated CBV (equivalent to Class 1 hemorrhage in adults, i.e. mild blood loss) in healthy children will only cause no more than mild tachycardia [33]. By excluding patients with blood loss above 10%, which may possible influence hemodynamic, untoward effects of blood loss on COP were probably eliminated. Excessive provision of fluid will decrease COP_p_ and also decrease COP_i_ subsequent to increased filtration of fluid into the interstitium. Approximately 80% of the patients received fluid in excess of basic needs during the surgical procedure, but they were not given fluids during several hours before surgery and it is likely that the fluid balance was within normal levels at the time of sampling. Therefore, it is reasonable to assume that our data on COP represented normal physiological values.

We found a significant increase in ΔCOP from 2 to 7 years. Such increase will favor transport of fluid into the capillaries and concurrent reduced absorption of fluid by the lymphatic system in order to preserve homeostasis. Earlier studies have shown that a local rise in COP_p_ will counteract edema formation in tissue with reduced blood flow [34], as observed in our study, where COP_p_ and lymph drainage is thought to be important. A reduced ΔCOP for 8–10 years is probably due to higher COP_i_ compared to a net increase in COP_p_ with age. Whether this decline in delta COP is caused by a hydrostatic effect of patients being taller or a result of few observations remains uncertain.

COP_i_ is, together with interstitial hydrostatic pressure, COP_p_ and lymph flow, important regulators of interstitial fluid balance. These factors may also counteract edema formation in situations with hyperfiltration. Recording of interstitial fluid pressure (P_i_) in relation to COP_p_ and COP_i_ would have been useful, but this was not feasible under the current clinical conditions.

In a revision of the traditional form of Starling`s principle, Levick and Michel advocate that COP_i_ in the “global” tissue at a distance from the capillary has less effect on capillary filtration than previously anticipated due to the presence of a semipermeable endothelial glycocalyx layer (EGL) built up of glycoproteins and glycosaminoglycanes [35]. The EGL will result in a COP gradient into the capillary that is higher than that calculated from ΔCOP. A recent review by Woodcock and Woodcock emphasizes that maintenance of the integrity of EGL might be of significance for fluid resuscitation to support the circulation in high filtration states [36]. The conclusions of a reduced importance of the COP_i_ in fluid filtration originates from experiments with high filtration pressures, whereas studies in mesenteric capillaries of rats suggests that the oncotic transcapillary pressure gradient is highly filtration dependent and that COP_i_ is close to COP of EGL at normal filtration pressures [37]. As recently discussed by Wiig and Swartz [38], with these reservations, we may still conclude that COP_i_ is of major importance for normal fluid filtration.

The present study has some limitations. All subjects were under general anesthesia and, therefore, may have had altered homeostasis, which could impinge upon normal COP values, although maintenance of anesthesia with propofol is associated with minimal fluid extravasation [39]. Due to our protocol, additional implantation of wicks was not accepted when traumatic bleeding occurred, which reduced the number of simultaneous determination of COP in plasma and IF. Plasma COP should optimally be sampled at the same time as harvesting the wicks, not only before implantation. This is especially important for patients with traumatic bleeding over 10% of CBV since a fall in hematocrit probably would alter COP_p_ as well as COP_i_. Due to our protocol, and the wish to avoid harmful procedures in non-therapeutic research, it was not possible to sample blood after discontinuation of anesthesia.

In conclusion, children between 2 and 10 years of age have plasma COP values similar to adults, with raised COP_p_ and COP_i_ with increasing age. These findings are important to acknowledge, since small alterations in the pressure gradient over the capillary membrane can cause substantial fluid shifts. Increasing knowledge of COP in both health and disease and the influence of crystalloids, colloids and diuretics on COP may be beneficial optimizing clinical care both in adults and the pediatric population.

## Supporting Information

S1 Checklist CONSORT Checklist. (PDF)

S1 Ethics approval Ethical Confirmation from the Regional Committee for Medical and Health Research Ethics, Western Norway. (PDF)

S1 Fig Weight and standard deviation according to Norwegian growth charts. 2 SD equals 97.7 percentile and -2 SD equals 2.3. percentile.
(PDF)

S1 Protocol Trial protocol in Norwegian. (PDF)

S2 Protocol Trial protocol in English. (PDF)
