# Supplementary material for: Interstitial Fluid Colloid Osmotic Pressure in Healthy Children
Source: PLoS One. 2015 Apr 8;10(4):e0122779. doi: 10.1371/journal.pone.0122779 (PMC4390290; doi:10.1371/journal.pone.0122779)
Supplement: S1 Ethics approval — (PDF) [file pone.0122779.s002.pdf]

To whom it may concern

Your ref	Our ref	Date
	135.06	12.09.2014

Confirmation;

I hereby confirm that the project "Interstitial fluid colloid osmotic pressure in healthy children", by project manager Ansgar Berg, is reviewed and approved (29.06.2006) by the Regional Committee for Medical and Health Research Ethics, Western-Norway.

Best regards

Øyvind Straume
Committee secretary
