# Supplementary figures and images for: Interstitial Fluid Colloid Osmotic Pressure in Healthy Children
Source: PLoS One. 2015 Apr 8;10(4):e0122779. doi: 10.1371/journal.pone.0122779 (PMC4390290; doi:10.1371/journal.pone.0122779)

## Girls Weight and Standard Deviation

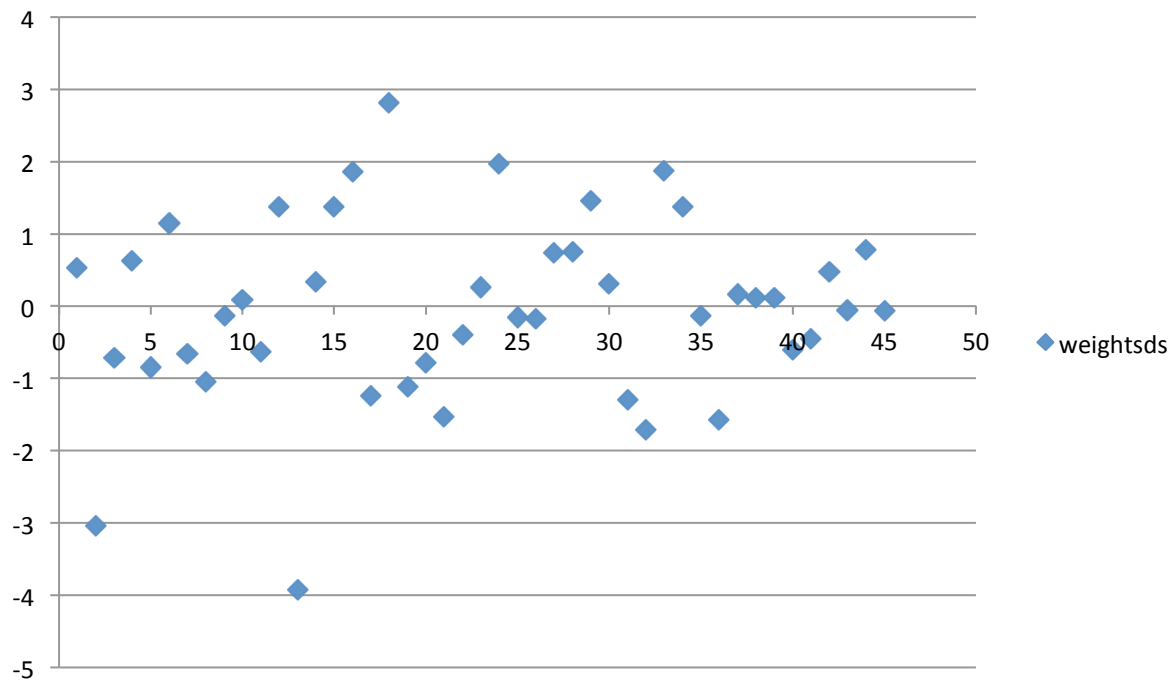

## Boys Weight and Standard Deviation

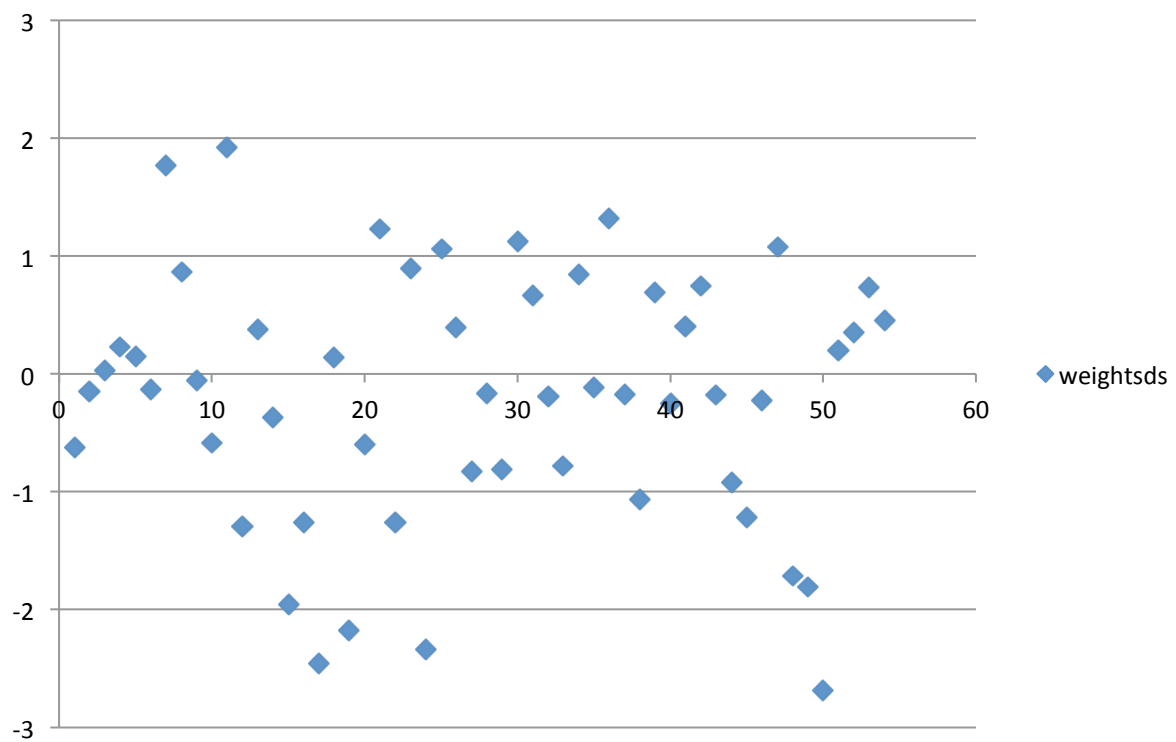

Supplement: S1 Fig — 2 SD equals 97.7 percentile and -2 SD equals 2.3. percentile. (PDF) [file pone.0122779.s003.pdf]
