# Supplementary material for: Interstitial Fluid Colloid Osmotic Pressure in Healthy Children
Source: PLoS One. 2015 Apr 8;10(4):e0122779. doi: 10.1371/journal.pone.0122779 (PMC4390290; doi:10.1371/journal.pone.0122779)
Supplement: S1 Protocol — (PDF) [file pone.0122779.s004.pdf]

Protokoll

Måling av kolloid osmotisk trykk og proteindistribusjon i plasma og interstitiell væske hos barn

Prosjektansvarlig: [2,4]Ansgar Berg (Overlege, Professor Dr. med)

Medarbeidere: [1,3]Torbjørn Nedrebø (Lege i spesialisering, Dr.med)., [3]Helge Wiig (Professor Dr. med), [1]Per Anders Hunderi (Overlege), [5]Shashi Gulati (Overlege), [4]Hans-Jørgen Guthe (Lege i spesialisering)

1) Haukeland Universitetssykehus, Kirurgisk Serviceklinikk
2) Institutt for klinisk medisin, Seksjon for pediatri, Universitetet i Bergen
3) Institutt for Biomedisin, Seksjon for fysiologi, Universitetet i Bergen
4) Haukeland Universitetssykehus, Barneklinikken
5) Haukeland Universitetssykehus, Klinikk for Hode-Hals, Øre-Nese-Hals avd.

BAKGRUNN

Væskevolum og fordeling av kroppsvann hos voksne og barn

Circa 60% av kroppsvekten utgjøres av vann hos et voksent menneske. Hos nyfødte under 1 år er vanninnholdet nærmere 80% av kroppsvekten. Etter første leveår synker vanninnholdet og en regner at ved 9 års alder er vanninnholdet tilnærmet som hos et voksent individ. Fra et funksjonelt og klinisk synspunkt tenker vi oss vannvolumet delt opp i 2 adskilte væskerom: 1) Intracellulært væskevolum (ICV) og 2) Ekstracellulært væskevolum (ECV). Grovt regnet finnes 2/3 av kroppens vannvolum intracellulært og de resterende 1/3 fordeles ekstracellulært. Det ekstracellulære væskevolum inndeles i 2 komponenter; 1) Intravasale væskevolum (= plasmavolum som utgjør 25% av ECV) og 2) Ekstravasale væskevolum (= interstitielt væskevolum, utgjør 75% av ECV eller ca 12-15% av kroppsvekten hos en voksen). Hos barn er det økte væskevolum per kroppsvekt fremfor alt lokalisert som ektracellulært vannmengde sett i forhold til et voksent individ. Reduksjonen av kroppsvannets del av den totale kroppsvekt under de første 10 årene skyldes derfor i hovedsak en reduksjon av ekstracellulært vanninnhold. Dette utgjør hos nyfødte omkring 40% av kroppsvekten og den relative mengden synker til 20% ved puberteten. Plasmavolumet utgjør ca 5% av kroppsvekten i alle aldersgrupper.

Væskebalanse

Reguleringen av det ekstracellulært volum er av sentral betydning for liv og helse. Sviktende regulering – som ved hypovolemi – vil i verste fall føre til hypovolemisk sjokk og kan være livstruende. Omvent; grov hypervolemi – som ved eksessiv infusjonsterapi – vil kunne føre til hjertesvikt, lungeødem og fare for liv. Ikke forbausende ligger det innebygget i organismen robuste mekanismer som regulerer og stabiliserer det ekstracellulært volum tross hyppige forekommende, livssituasjonsbetingede store svingninger i salt- og vann inntak – og tap. Disse reguleringsmekanismene sørger til enhver tid for best mulig situasjonsadekvat salt- og væskebalanse, stabilisering av osmolariteten i vevsvæsken og adekvat blodtrykk. Reguleringen forekommer som et resultat av tett samspill mellom trykk- og volum reseptorer i sentrale sirkulasjonsavsnitt, nyrene, det autonome nervesystemet og endokrinologiske systemer (aldosteron og Antidiuretisk hormon).

Regulering av væsketransport mellom plasma og interstitiet foregår ved at væskefasene kommuniserer med hverandre på kapillærnivå. Forutsetningen for væsketransport over mellom disse fasene er at ved at kapillærsengen er permeabel overfor vann og krystalloider. Væskeutvekslingen mellom plasma og interstitiell væske skjer ved trykkfiltrasjon og reabsorpsjon. Kapillærsengen er semi-permeabel for plasma proteiner og de fleste av disse blir ikke trykkfiltrert, men holdes tilbake i sirkulasjonen. Trykkgradientene for filtrasjon og reabsorpsjon over kapillærmembranen bestemmes av summen av de hydroststaiske og kolloide osmotiske trykkgradienter i de ulike avsnitt av kapillærsengen. Transkapillær transport av væske (Jv) er bestemt av de transkapillære hydrostatiske (P) og kolloid osmotiske trykk (COP) slik de er beskrevet i likningen (Starlings likning):

$$Jv= \quad CFC\ ((Pc\text{-}Pif)\ \text{-}\sigma(COPc\text{-}COPi)) = CFC \cdot \Delta P = \text{Lymfeflow}$$

hvor CFC er kapillærfiltrasjonskoeffisient (vannpermeabilitet), σ er refleksjonskoeffisienten for plasma proteiner, ΔP er netto trykkgradient over kapillærene og ”c” er og ”i” er henholdsvis kapillær og interstitiell væske. Normalt er det en netto filtrasjon av væske over kapillærmembranen som vil balanseres av en tilsvarende lymfedrenasje (lymfeflow) slik at steady-state oppnås. Enhver forandring i faktorene av Starlings likning, de hydrostatiske trykkene, de colloid osmotiske trykk eller lymfeflow vil påvirke transkapillær væsketransport og derfor både plasma volum og interstitiellt volum. Det er viktig å være klar over at parametrene i Starlings likning ikke er statiske, men endringer i en parameter ofte medfører kompensatoriske endringer i de andre parametrene. For eksempel vil en økning i kapillær trykk (Pc) eller fall i COPp føre til netto transport av væske fra plasma til interstitiet som medfører økt interstitielt væsketrykk (Pi) og eller COPi (fortynning). Disse endringen ble første gang formulert av Guyton og ble kalt ”edema preventing mechanism” dvs mekanismer som motvirker ødemutvikling. Disse mekanismene er senere blitt studert og kartlagt i flere ulike dyremodeller og har dannet grunnlag for bedret forståelse av væskebalansen og væsketerapi ved ulike sykdomstilstander.

Transkapillære Colloid Osmotiske trykk (COP) hos voksne og barn

Metoder for sampling av interstitiell væske og måling av COP ble etablert allerede på 70-tallet ved Fysiologisk institutt, Universitetet i Bergen, og studier

av distribusjonen av plasmaproteiner og væskebalanse er blitt studert ved ulike sykdomstilstander med væskeretensjon, hypoproteinemi, hypotermi, nyresvikt, økt venetrykk, hos gravide med ødemtendens samt hos kvinner med premenstruell syndrom (PMS). Resultatene fra disse studiene har fått stor betydning for forståelsen av patofysiologien ved disse tilstandene og har vært viktige ved valg av behandling. Normal området for COPi for friske voksne er mellom 22 og 28 mmHg (gjennomsnitt 25 mmHg).
Hos nyfødte barn vil **COP i plasma** øke med økende gestasjonalder (ektremt premature lavest). **COP i plasma** hos friske nyfødte er også lavt i forhold til voksne, men COP plasma øker hos barn med økende alder (der den største endringen observeres i tidlige barneår). Betydningen av de lave **COP verdiene i plasma** hos friske barn (nyfødte, små barn) og syke barn (nefrotisk syndrom, blødninger, sepsis) er imidlertid ukjent da det er forholdet mellom COP i vevsvæsken og plasma (COPp-COPi) som bestemmer væskebalansen over kapillærmembranen. Den kliniske verdien av plasmamålinger av COP hos barn har derfor vært begrenset og væsketerapi på bakgrunn av de målte verdier har basert seg på ekstrapolering fra data hos voksne. Data for **COP i interstitiet** hos barn **mangler imidlertid helt** og skyldes i hovedsak manglende metoder til å sample interstitialvæske. Kjennskap til ***COP i vevsvæske*** er imidlertid viktig hos friske barn fordi verdiene vil være til nytte ved væsketerapi hos kritisk syke barn. I dag foreligger ingen normalverier for COP i interstitiet hos friske barn, og dermed kan man heller ikke nyttiggjøre seg av COP plasma verdier hos kritisk syke barn og prematurer. Klarer man å etablere sikre og gode normalverdier på COPi hos friske barn vil man ha et godt utgangspunkt for væsketerapi hos kritisk syke barn.

BETYDNING

Analyse av proteininnhold og COP i interstitial væske gir verdifull informasjon om væskebalansen. Kunnskap om væskebalansen hos barn er vesentlig innen de fleste anestesiologiske, kirurgiske og pediatriske virkeområder. Bedret forståelse av væskebalansen øker våre muligheter til å gi en adekvat væsketerapi eller ikke minst unngå en inadekvat eller unødig væske terapi. Adekvat væsketerapi vil ofte være avgjørende for en pasientens velbefinnende og i noen situasjoner også være helt avgjørende for om pasienten blir frisk eller overlever. Man vet albumin

utgjør 80% COP i plasma hos friske individer, men kun 17% hos kritisk syke voksne. Tilsvarende data mangler hos barn. Albuminholdige væsker (kolloider) blir ofte anvendt for å korrigere hypo-albuminemi hos voksne intensivpasienter uten at det er vist effekt på mortalitet. Lignende data for barn mangler helt og kunnskap om normalverdier for COP i plasma og interstitiet vil kunne har stor betydning for valg av behandling ved væskesubstitusjon og hypoproteinemi tilstander (nefrotisk syndrom, malign sykdom ol).

TIDSPERIODE

Studien vil starte opp 1. september 2006, og man regner med å kunne avslutte studien 31 desember 2007. Man vil avslutte studien før tiden om kravet til 100 pasienter oppfylles før.

MÅLSETTINGER OG HYPOTESER

Prosjektet ønsker å etablere metoder for sampling og måling av collid osmotisk trykk (COPi) i vevsvæske **hos barn**. Det primære målet med studien er å kartlagge normalverdier COPi hos friske barn i aldersgruppen 2-10 år. Det foreligger per i dag ingen data vedrørende verdier av COPi verken hos friske eller syke barn. Vi har i den forbindelse fem hovedhypoteser:

1) Det er mulig å sample vevsvæske ved vekemetoden og måle COPi (og proteinfraksjoner) hos barn i aldersgruppen 2-10 år.
2) Tiden for at likevekt oppnås ved veke-metoden er tilsvarende som hos voksne (60 min) (forutsetningen for at verdiene er reelle).
3) COPi er aldersavhengig og korrelerer godt med de aldersavhengige verdiene for COP i plasma.
4) COPi i ankel er korrelert til høyde og forskjellen på COPi i ankel og thorax vil derfor øke med økende høyde (Hydrostatisk trykk i underekstremiteter er avhengig av gravitasjonskrefter og derved vil økt høyde kunne gi økt filtrasjon av proteiner i subcutant vev i ankel).
5) Forholdet mellom Albumin og Totalprotein (albumin/totalprotein ratio) i vevsvæsken øker med økende alder (verifisere de kliniske observasjoner

under væsketerapi og dyre eksperimentelle data som tilsier kapillærsengen hos de minste barna ”lekker” mest).

PASIENTER

Populasjon vil rekrutteres fra ”friske” pasienter som innlegges til elektiv kirurgisk behandling (tonsillectomi, paracentese/dren-innleggelse, adenotomi) ved Klinikk for Hode-Hals, avdeling for Øre-Nese-Hals, Haukeland Universitetssykehus. Det behandles årlig ca 500-600 (10-15 per uke i 40 uker) barn med disse diagnosene ved Haukeland Universitetsykehus. Storparten av pasienten vil være i aldersgruppen 2-5 år. De fleste pasienter vil være i narkose mellom 60 og 90 minutter i forbindelse med den kirurgiske prosedyre.

Protokoll. Det vil bli tatt 1 venøs blodprøve (1 - 2 ml fra veneflon) for bestemmelse av plasmaprotein og COP. Samtidig vil 2 ”veker” bli innført subcutant (ankel + thorax) for bestemmelse av proteininnhold og COP i vevsvæske (2-5 μl per veke). Begge prosedyrer vil bli gjennomført ***etter*** at barnet er lagt i narkose (kombinert fentanyl-propofol anestesi) og disse tas ut etter 60 (-90) minutter og ***før*** barnet ekstuberes / vekkes etter narkose og gjennomført kirurgisk inngrep. Etter at veken er fjernet vil man legge et vanlig plaster over innstikkstedet for veken. Barna vil smertelindres postoperativt etter gjeldene retningslinjer for det aktuelle inngrepet ved ØNH-poliklinikken. Under hele inngrepet og i det postoperative forløp vil blodtrykk, oksygenmetning, puls, respirasjonsfrekvens og evt. blødningmengde (fra ØNH-inngrepet) monitoreres. Prosjektet vil **ikke** ha noen innvirkning på inngrepets art, narkosevarighet eller post-operativ oppfølgning.

Inklusjonskriterier:	Informert samtykke fra foreldre<br>Alder 2 – 10 år
Eksklusjonskriterier:	Syndromer (ex Down Syndrom, Turner)<br>Kroniske sykdommer (ex Hjerte- eller Nyresykdommer)<br>Blødning fra kirurgisk inngrep mer enn 10% av totalt blodvolum under operasjonen

Antall pasienter som skal inkluderes vil være 100 barn totalt (10 i hver aldersgruppe (2-3, 3-4, 4-5, 5-6, 6-7, 7-8, 8-9, 9-10 år) n=80. Hos 20 barn der narkosevarighet antas å vare > 90 minutter vil *begge vekene* settes subcutant i ankelen der 1 veke fjernes etter 60 minutter og den andre etter 90 minutter (undersøke ekvillibreringstid for vekemetoden hos barn)

INFORMASJON, INNHENTING AV SAMTYKKE OG REKRUTTERING AV PASIENTER

Foresatte til barn som skal til mindre inngrep ved Øre-Nese-Hals poliklinikken ved Haukeland sykehus vil etter at de har fått innkallelse fra sykehuset til operasjon, få tilsendt ett informasjonsskriv (se vedlegg 1.). I informasjonsskrivet ligger en forespørsel om å deltagelse i studien. Det gis anledning for foresatte til å ringe prosjektansvarlig (Dr Nedrebø / Dr Berg) dersom utfyllende informasjon ønskes eller spørsmål dukker opp. Sammen med informasjonen sendes en samtykkeerklæring som de foresatte må underskrive på og sende inn til prosjektansvarlig. Ca. en uke før operasjon vil man kontakte de foresatte som har sagt seg villig til å la sitt barn delta i studien, pr telefon for å ha bekrefte at de fortsatt er sier seg villig, svare på eventuelle spørsmål og planlegge praktiske forhold ved inklusjon.

METODER

Proteininnhold og COP i plasma bestemmes fra den venøse blodprøven. Vevsvæske til COPi målingene samles ved at man syr inn **to** veker i subcutant vev (figur 1a). Vekene er ca 2 mm tykke, og består av ca 300 nylonfibre som er sterilisert med gamma-stråling. Vekene vil settes inn på høyre legg og høyre thorax etter at barna er lagt i narkose, og etter at huden er vasket med 0.5% klorhexidin. Under sterile forhold, og ved hjelp av en nål sys 30-40 mm av veken inn subcutant i huden (figur 1a). Veken klippes i begge ender slik at ca 10 mm av veken blir liggende utenfor huden. Over dette legges en steril plastikk film. Veken blir liggende 1 time (eller 90 min hos 20 pasienter) før den fjernes og legges i et sentrifugerør med mineralolje (figur 1b). Tuben sentrifugeres, og den interstitielle væsken samlet i veken vil legge seg på bunnen av røret, og kan videre analyseres (figur 1c). Ved hjelp av denne metoden vil en få ut 2-5 μl per veke og deler av denne væsken (1-2 μl) vil brukes til måling av COP ved hjelp av

et kolloid osmometer (figur 1d) designet for små prøvevolum (1 mikroliter). Resterende volum vil analyseres for proteininnhold og distribusjon av makromolekyler ved hjelp av et HPLC system.

Figur 1.

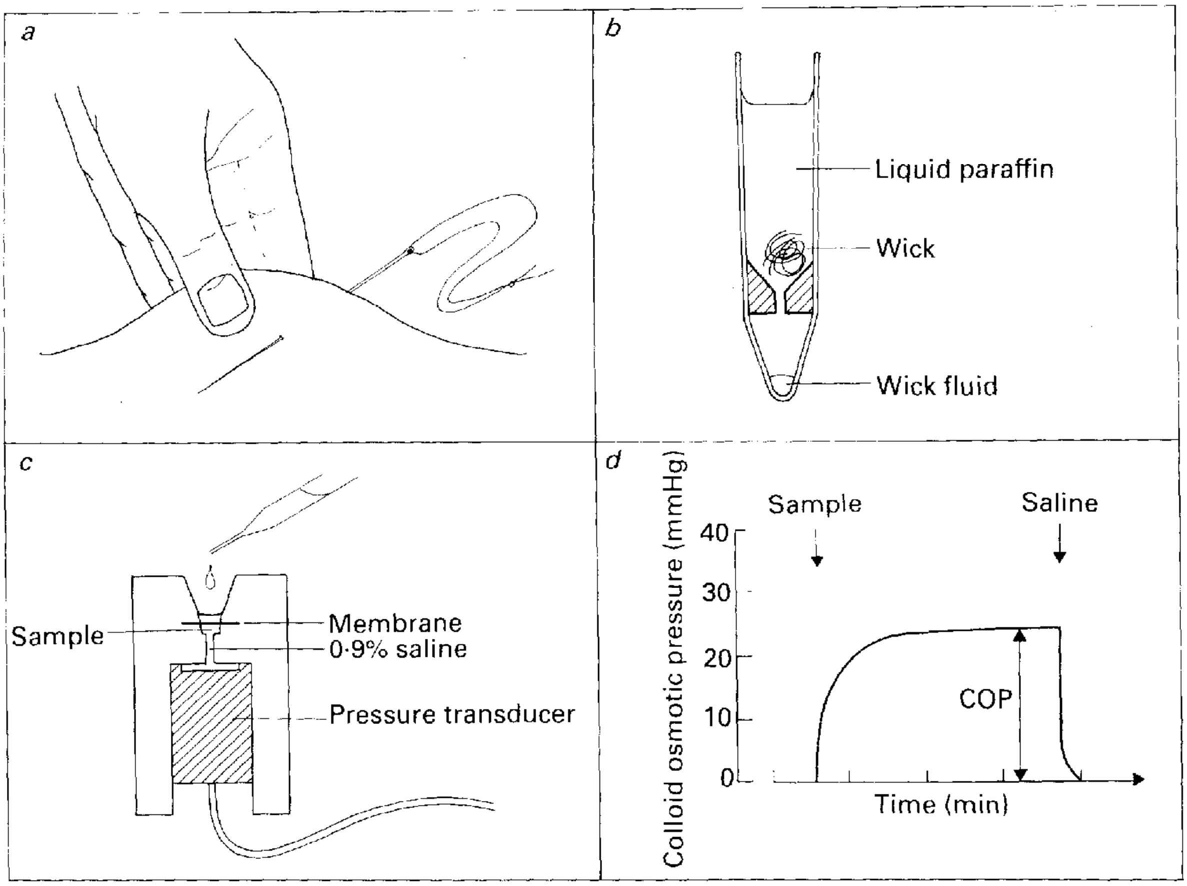

MONITORERING/KVALITETSIKRING

Det er ikke opprettet noen ekstern og uavhengig monitor i denne studien (ikke krav om dette). Prosjektansvarlig vil være ansvarlig for at protokollen følges og at korrekte data samles inn. Uventede hendelser vil rapporteres og all dokumentasjon vil lagres. Jevnlige møter vil arrangeres for deltagerne i prosjektet slik at prosjektets fremdrift evalueres og der eventuelle problemer i studien kan tas opp fortløpende.

STATISTIKK

Power beregning ikke aktuelt pga at studien i hovedsak er rent deskriptiv. Data vil bearbeides ved vanlige deskriptive statistiske metoder.

FORSKNINGSETIKK OG SIKKERHET

Protokollen med informasjonsskriv til foreldrene legges frem for Regional etisk komite for medisinsk forskningsetikk og Personvernombudet ved NSD. Prosjektleder er ansvarlig for all pasient anonymitet ved datalagring og analyse. Deltagerne vil avgi skriftelig samtykke og er klar over at de kan trekke seg fra deltagelse på hvilket som helst tidspunkt uten begrunnelse og uten at det skal ha noen betydning for behandlingen ellers i sykehuset. Vi har i informasjonsskrivet beskrevet og forklart hvordan selve studien vil foregå og om risiko ved deltagelse. Metoden vi bruker er velkjente, trygge og vil ikke påføre barnet noe smerte (generell anestesi). Vekemetoden brukes fordi denne er enkel å utføre, nesten smertefri og fri for komplikasjoner. I forsøk med gravide kvinner ble over 300 kvinner undersøkt uten en eneste komplikasjon. Studien vil ikke påvirke den kirurgiske behandlingen, narkosetype/ -varighet, eller den postoperative overvåkning.

FORSKERGRUPPES KOMPETANSE OG UTSTYR

Prosjektleder har etter endt doktorgrad vært tilknyttet Institutt for Biomedisin, seksjon for fysiologi, og er en del av en større forskningsgruppe i området **sirkulasjon og væskebalanse**. Deler av gruppen er blitt evaluert av internasjonale utvalg de siste årene. Dette har blant annet resultert i tildeling av midler fra EU-programmet Training and Mobility of Researchers (TMR) (1998-2002) og gruppen fikk status som Marie Curie training site (2001-2004). Gruppens forskning ble vurdert som ”very good to excellent” av et internasjonalt panel som i år 2000 (på oppdrag fra NFR) evaluerte biomedisink forskning i Norge. Disse evalueringene viser etter vår mening at forskningsarbeidet i gruppen, der flere av prosjekt medarbeiderne har hatt eller har sin tilknytning, er på et høyt internasjonalt nivå. Prosjektet viderefører også et samarbeidet mellom basalfagene og den kliniske forskningen ved å bruke klassiske fysiologiske metoder i viktige kliniske problemstillinger. Alt utstyr og alle metoder som vil anvendes i prosjektet er tilgjengelig og i daglig bruk ved Inst. for Biomedisinske fag, seksjon for fysiologi. Innsamling av data vil foregå ved prosjektmedarbeiderne (Berg, Nedrebø og Guthe) som en del av den kliniske rutinen ved Haukeland Universitetssykehus.

LITTERATURHENVISNINGER

1) Friis-Hansen B. Acta Paediatr 1954
2) Morissette MP. Can Med Assoc J 1977
3) Fadnes & Aukland, *Microvasc Res*, 1977
4) Fadnes HO, *Scand J Lab Clin Invest*, 1975
5) Miki K, J UOEH, 1983
6) Haneda et al,*J Cardiovasc Surg*, 1987
7) Fadnes et al, *Scand J Lab Clin Invest*, 1986
8) Leslie GI, Aust Paediatr J 1987.
9) Fadnes HO, *Scand J Lab Clin Invest*, 1976
10) Øian et al, Br *J Obstet Gynaecol*, 1986
11) Tollan et al, Acta *Obstet Gynecol Scand*, 1990
12) Blackwell MM, J Extra Corpor Technol 1994
13) Blunt et al, *Anaesthesia*, 1998
14) Øian et al, *Br J Obstet Gynaecol*, 1985
15) Sussmane JB, Crit Care 2001.
16) Wiik H AJP 2005.
