# Supplementary material for: Interstitial Fluid Colloid Osmotic Pressure in Healthy Children
Source: PLoS One. 2015 Apr 8;10(4):e0122779. doi: 10.1371/journal.pone.0122779 (PMC4390290; doi:10.1371/journal.pone.0122779)
Supplement: S2 Protocol — (PDF) [file pone.0122779.s005.pdf]

Protocol

Translated form the Norwegian original protocol

Measurement of colloid osmotic pressure and protein distribution in plasma and interstitial fluid in children

Principal Investigator: [2,4]Ansgar Berg (Consultant, Professor Dr. med)

Co-workers: [1,3]Torbjørn Nedrebø (Doctor of specialization, Dr. med), [3]Helge Wiig (Professor Dr. med) [1]Per Anders Hunderi (Consultant), [5]Shashi Gulati (Consultant), [4]Hans-Jørgen Guthe (Doctor of specialization)

1) Haukeland University Hospital, Surgical Service Clinic
2) Institute of Clinical Medicine, Department of Paediatrics, University of Bergen
3) Institute of Biomedicine, Department of Physiology, University of Bergen
4) Haukeland University Hospital, Department of Paediatrics
5) Haukeland University Hospital, Division of Head-Neck, Ear-Nose-Throat dept.

BACKGROUND

Liquid volume and distribution of body water in adults and children

Approximately 60% of an adult´ body weight consists of water. In neonates under 1 year of age, the water content is close to 80% of body weight. After the first year of life, the water content decreases and by 9 years of age the water content is approximately the same as for adults. From a functional and clinical point of view, the water volume can be divided into two separate fluid compartments: 1) Intracellular fluid volume (ICV) and 2) extracellular fluid volume (ECV). Roughly two thirds of the body's water volume is located intracellular and the remaining third is distributed extracellular. The extracellular fluid volume is divided into two components; 1) intravascular fluid volume (= plasma volume represents 25% of ECV) and 2) extravascular fluid volume (= interstitial fluid volume, representing 75% of ECV or about 12-15% of the body weight of an adult). Compared to adults, the increased fluid volume per body weight is primarily localized as extracellular fluid in children. The reduction of body water, compared to total body weight during the first 10 years of life, is therefore primarily due to a reduction of extracellular water content. This amount constitutes approximately 40% of the body weight in neonates, and the relative amount decreases to 20% at puberty. Plasma volume represents about 5% of body weight in all age groups.

Fluid balance

The regulation of the extracellular volume is of central importance to life and health. Failing regulation - as in hypovolemia - will at worst lead to hypovolemic shock and may be life threatening. On the other hand, gross hypervolemia - as with excessive infusion therapy - can lead to heart failure, pulmonary edema and potentially death. Robust mechanisms regulate and stabilize the extracellular volume, despite frequently occurring wide fluctuations in fluid losses and in salt and water intake. These regulatory mechanisms ensure, at all times, the best possible situation: Adequate salt and fluid balance, stabilization of the osmolality of interstitial fluid and adequate blood pressure. The regulation occurs as a result of the close interaction between pressure and volume receptors in the central circulation, kidneys, autonomic nervous system and endocrine systems (aldosterone and vasopressin).

Regulation of fluid transport between plasma and the interstitium takes place by communication between the fluid phases on a capillary level. Fluid transport over the capillaries requires a vessel wall that is permeable to water and crystalloids. Fluid exchange between plasma and interstitial fluid occurs by filtration due to hydrostatic pressure and absorption. The capillary wall is semi-permeable to plasma proteins, and most of these will not be pressure filtered, but be retained in the circulation. The pressure gradient for filtration and reabsorption of the capillary membrane is determined by the sum of the hydrostatic pressure, and the colloid osmotic pressure in the various sections of the capillary. Transcapillary transport of fluid (Jv) is determined by the trans-capillary hydrostatic (P) and colloid osmotic pressure (COP) as described in equation (Starlings equation):

$$Jv = CFC\,((Pc - Pi) - \sigma\,(COPc - COPi)) = CFC \cdot \Delta p = \text{Flow of lymph}$$

CFC is capillary filtration coefficient (water permeability), σ is the reflection coefficient for plasma proteins, Δp is the net pressure gradient across the capillaries and "c" and "i" are the capillary and interstitial fluid respectively. Normally, there is a net filtration of fluid across the capillary membrane, which will be balanced by a corresponding lymph drainage (flow of lymph) in order to achieve steady-state. Any change in the factors of Starlings equation, the hydrostatic pressures, the colloid osmotic pressures or flow of lymph, will affect transcapillary fluid transport and therefore both the plasma volume and the interstitial volume. It is important to note that the parameters of Starlings equation are not static, but changes in one parameter will often lead to compensatory changes in the other parameters. For example, an increase in capillary pressure (Pc) or fall in COPc, will lead to net transport of fluid from the plasma to the interstitium, resulting in increased interstitial fluid pressure (Pi) and/or a decrease in COPi (dilution). These changes were first postulated by Guyton and were called "edema preventing mechanism", i.e. mechanisms that counteract edema development. These mechanisms have subsequently been studied and mapped in several animal models, and has formed the basis for improved understanding of fluid balance and fluid therapy in various disease states.

Transcapillary Colloid Osmotic Pressure in adults and children
Methods for sampling interstitial fluid and measurement of COP were established in the 1970s at the Department of Physiology, University of Bergen. Studies of the distribution of plasma proteins and fluid balance have been examined for various disease states with fluid retention, hypoproteinemia, hypothermia, kidney failure, increased venous pressure, pregnancy with edema, and in women with premenstrual syndrome. The results of these studies have been of great importance for the understanding of the pathophysiology of these conditions, and have been important in the choice of treatment. Normal range for COPi for healthy adults is between 22 and 28 mmHg (mean 25 mmHg).
For newborn babies, COP in plasma would increase with increasing gestational age (extreme premature babies have lowest COP). COP in the plasma of healthy newborns are also low compared to adults, but COP in plasma increases with increasing age (especially in early childhood). The significance of the low COP values in plasma of healthy children and sick children (nephritic syndrome, traumatic bleeding, sepsis) is unknown because it is the relationship between COP in interstitial fluid and plasma (COPp-COPi), which determines the water balance of the capillary membrane. The clinical value of plasma COP in children has therefore been limited, and fluid therapy has been based on extrapolation from data in adults. COP data from the interstitium in children, however, lacks entirely due to lack of methods to sample interstitial fluid. Recognition of COP in interstitial fluid in healthy children is nevertheless important to address because this knowledge will benefit fluid therapy in critically ill children. Today, normal values for COP from interstitial fluid in healthy children do not exist, and thus the use of COP plasma levels in critically ill children and premature is of limited value. Establishing normal values for COP in interstitial fluid in healthy children is therefore important to understand fluid therapy in critically ill children.

SIGNIFICANCE

Analysis of protein content and COP in interstitial fluid provides valuable information regarding fluid balance. Knowledge of fluid balance in children is essential in most anesthetic, surgical and pediatric work areas. Improved understanding of fluid balance increases our ability to provide adequate fluid therapy, or at least avoid inadequate or excessive fluid therapy. Adequate fluid therapy is often critical to a patient's wellbeing and is in some situations essential for survival. Albumin accounts for 80% COP in the plasma of healthy individuals, but only 17% in critically ill adults. Similar data are lacking in children. Albumin solutions (colloids) are often used to correct hypoalbuminemia in adult intensive care units (ICU), patients without evidence of adverse effect on mortality. Similar data for children are missing completely and knowledge of normal values for COP in plasma and in the interstitium will have great significance for the choice of treatment for fluid substitution and hypoproteinemia states (nephrotic syndrome, malignant disease, etc.).

TIME PERIOD

The study will begin on 1 September 2006 and we expect to finish the study, 31 December 2007. If the aim of 100 patients is met before, the study will be completed earlier.

OBJECTIVES AND HYPOTHESIS

The project aims to establish methods for sampling and measurement of colloid osmotic pressure (COPi) in tissue fluid in children. The primary objective of this study is to find normal values of COPi in healthy children aged 2-10 years. There is currently no data on COPi either in healthy or sick children.
The project has five main hypotheses:

1) It is possible to sample tissue fluid by the wick method and measure COPi (and protein fractions) in children aged 2-10 years.
2) The time that equilibrium is achieved by the wick method is similar to that in adults (60 min.) (with the assumption that adult values of COP are reliable).
3) COPi is age-dependent and correlates well with the age-specific values for COP in plasma.
4) COPi ankle is correlated to the height, and difference between COPi in ankle and thorax will increase with increasing height (hydrostatic pressure in the lower limbs is dependent on gravitational forces and, thus, increased height may increase filtration of proteins in the subcutaneous tissue of the ankle).
5) The relationship between albumin and total protein (albumin / total protein ratio) in tissue fluid increases with increasing age (verify the clinical observations during fluid therapy and animal experimental data that indicate increased capillary-bed leakage with decreasing age).

PATIENTS

Population will be recruited from "healthy" patients admitted to elective surgery (tonsillectomies, tympanic membrane paracentesis, adenotomy) at the Department of Ear-Nose-Throat (ENT), Haukeland University Hospital. The hospital treats annually about 500-600 children (10-15 per week for 40 weeks per year) with these diagnoses. The majority of patients will be in the age group 2-5 years. Most patients will be under general anesthesia between 60 and 90 minutes in relation to the surgical procedure.

Protocol: There will be drawn one venous blood sample (1-2 ml from i.v. line) for determination of plasma protein and the COP of plasma. Two nylon "wicks" will be inserted subcutaneously (ankle + thorax) for determination of protein content and the COP in interstitial fluid (2-5 µl. per wick). Both procedures will be conducted after the child is under general anesthesia (combined fentanyl-propofol anesthesia) and the wicks will be removed after 60 (-90) minutes before the child is extubated/awakened from anesthesia. After removal of the wick a traditional band-aid will cover the site of insertion. All participants will be given analgesia postoperatively according to current guidelines at the ENT clinic. During the procedure and through the postoperative course, blood pressure, oxygen saturation, heart rate, respiratory rate and amount of bleeding will be monitored. The project will have no impact on the type of intervention, duration of anesthesia or post-operative follow-up.

**Inclusion criteria:**	Informed consent from parents<br>Age 2 - 10 years
**Exclusion criteria:**	Syndromes (ex. Down Syndrome, Turner)<br>Chronic diseases (ex Cardiovascular and Kidney Diseases)<br>Bleeding from surgical procedure more than 10% of total blood volume during surgery

Number of patients to be included will be a total of 100 children (10 in each age group (2-3, 3-4, 4-5, 5-6, 6-7, 7-8, 8-9, 9-10 years) n = 80. In 20 children where general anesthesia duration is expected to last> 90 minutes, both wicks will be inserted subcutaneously in the ankle where one week will be removed after 60 minutes and the second after 90 minutes (time for equilibrium for wick method in children)

INFORMATION, COLLECTION OF CONSENT AND RECRUITMENT OF PATIENTS

Parents or guardians of children admitted for smaller surgical intervention at the Ear-Nose-Throat clinic at Haukeland University Hospital will receive an information letter in advance of the surgery (see Appendix 1). In the information letter there is a request to participate in the study. There is an opportunity for parents or guardian to call the principal investigator/ co-worker (Dr Nedrebø / Dr Berg) if additional information is required. Together with the information letter there is a consent form that parents must sign and submit to the principal

investigator. About one week before surgery the children´s parents who have agreed to let their child participate in the study will be contacted (by telephone) to confirm participation. They will also have the opportunity to ask any question they might have related to the study.

METHODS

Protein content and COP in plasma will be determined from the venous blood sample. Interstitial fluid will be collected by sewing two wicks in to the subcutaneous tissue (Figure 1a). The wicks are about 2 mm thick and consist of about 300 nylon fibers, which are sterilized by gamma radiation. Wicks will be inserted above the right ankle and right lateral hemithorax after the patient is put under general anesthesia, and after the skin is washed with 0.5% chlorhexidine. Under sterile conditions, by means of the needle the 30-40 mm of the wick will be stitched subcutaneously under the skin (Figure 1a). The wick is trimmed at both ends leaving about 10 mm of wick outside the skin, covered with a sterile plastic film. The wick will remain under the skin for one hour (or 90 minutes in 20 patients) before it is removed and placed in a centrifuge tube with mineral oil (Figure 1b). The tube is centrifuged, and the harvested interstitial fluid is contained at the bottom of the tube. Tube containing interstitial fluid and mineral oil is frozen for further analyzes (Figure 1c). Using this method, it is possible to extract 2-5µl fluid per week, and 1-2 µl will be used for the measurement of the COP using a colloid osmometer (Figure 1d) designed for small sample volumes (1-2 µl). The remaining volume will be analyzed for protein content and distribution of macromolecules by means of an HPLC system.

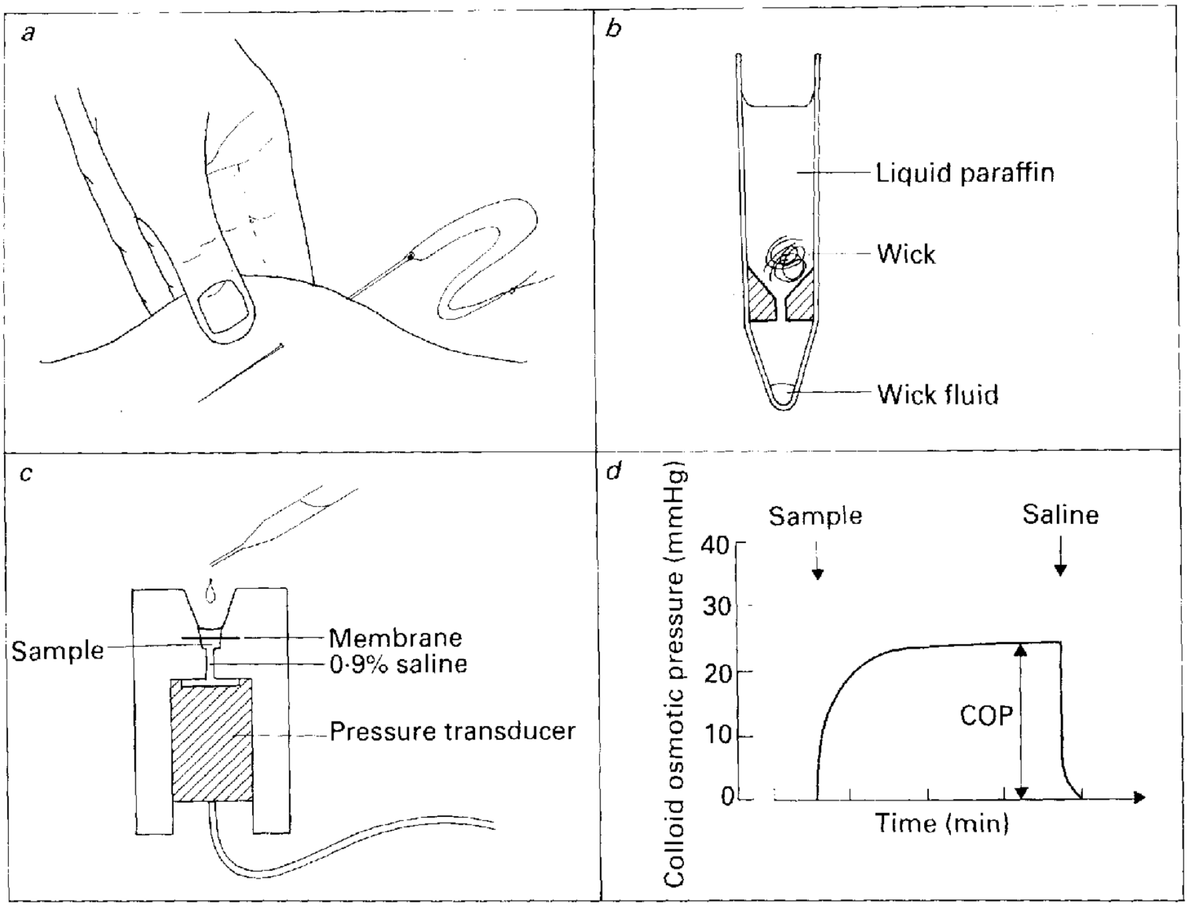

MONITORING / QUALITY GUARANTEE

There will be no external, independent monitor in this study (not required). The principal investigator will be responsible for ensuring that the protocol is followed and that appropriate data is collected. Unexpected events will be reported and all documentation will be stored. Regular meetings will be held for participants in the project so that the project's progress is evaluated and where possible, problems identified.

STATISTICS

Power calculation is not applicable because the study is essentially purely descriptive. Data will be processed by the usual descriptive statistical methods.

RESEARCH AND SAFETY

The protocol and parental information will be presented to Regional Ethics Committee for Medical Research Ethics and the Norwegian Social Science Data Services (NSD). The principal investigator is responsible for the patients' anonymity by data storage and analysis. Participants (parents) will submit a written consent before entering the study. They will be well informed about the study and aware of their right to withdraw from participation at any time without

giving any reason, knowing that this will not influence their use of hospital services. The participants will be explained how and when the study is to take place and the risks involved with participation. The method used in this study is well known, safe and will not cause the child any pain (general anesthesia). The wick method for collecting interstitial fluid is preferable because it is easy to perform, almost painless and free of complications. In experiments with pregnant women (over 300 women examined) no single complication occurred. The study will not affect the planned surgical treatment, anesthesia type / duration, or post-operative monitoring.

THE RESEARCH GROUPS EXPERTISE AND EQUIPMENT

The principal investigator has, after completing his PhD, been affiliated with the Department of Biomedical Sciences, Section of Physiology, and is part of a larger research group in the area of circulation and fluid balance. In recent years, different international committees have evaluated parts of the group. These evaluations have resulted in the allocation of funds from the EU program "Training and Mobility of Researchers" (TMR) (1998-2002), and the group has also acquired the status of Marie Curie training site (2001-2004). The group's research was rated as "very good to excellent" by an international panel in 2000 (on behalf of the Norwegian Board of Science) that evaluated biomedical research in Norway. These evaluations state that these research groups, where several of the co-workers have had or have their affiliation, are at a high international level. The project is also important for the collaboration between basic science and clinical research, using classical physiological methods in important clinical problems. All equipment and methods that will be used in the project are available and in daily use by Department of Biomedical Sciences, Section of Physiology. Collection of data will be conducted by project staff (Berg, Nedrebø and Guthe) as part of the clinical routine at Haukeland University Hospital.

LITERATURE REFERENCES

1) Friis-Hansen B. Acta Paediatr 1954
2) Morissette MP. Can Med Assoc J 1977
3) Fadnes & Aukland, *Microvasc Res*, 1977
4) Fadnes HO, *Scand J Lab Clin Invest*, 1975
5) Miki K, J UOEH, 1983
6) Haneda et al,*J Cardiovasc Surg*, 1987
7) Fadnes et al, *Scand J Lab Clin Invest*, 1986
8) Leslie GI, Aust Paediatr J 1987.
9) Fadnes HO, *Scand J Lab Clin Invest*, 1976
10) Øian et al, Br *J Obstet Gynaecol*, 1986
11) Tollan et al, Acta *Obstet Gynecol Scand*, 1990

12) Blackwell MM, J Extra Corpor Technol 1994

13) Blunt et al, *Anaesthesia*, 1998

14) Øian et al, *Br J Obstet Gynaecol*, 1985

15) Sussmane JB, Crit Care 2001.

16) Wiik H AJP 2005.
